# Development of quality indicators for low-risk labor care provided by midwives using a RAND-modified Delphi method

**DOI:** 10.1186/s12884-017-1468-4

**Published:** 2017-09-22

**Authors:** Kayo Ueda, Shosuke Ohtera, Misato Kaso, Takeo Nakayama

**Affiliations:** 1 Department of Health Informatics in the School of Public Health, Kyoto University, Yoshida Konoe-cho, Sakyo-ku, Kyoto, 606-8501 Japan; 2 School of Nursing, Faculty of Health Sciences, Morinomiya University of Medical Sciences, 1-26-16 Nankokita, Suminoe-ku, Osaka, 559-8611 Japan

**Keywords:** Low-risk labor, Quality indicator, Clinical practice guidelines, RAND-modified Delphi method

## Abstract

**Background:**

In childbirth, most deliveries are low-risk, defined as spontaneous labor at full term without special high-risk facts or complications, especially in high-resource countries where maternal and perinatal mortality rates are very low. Indeed, the majority of mothers and infants have no serious conditions during labor. However, the quality of care provided is not assured, and performance may vary by birthing facility and provider. The overuse of technology in childbirth in some parts of the world is almost certainly based on assumptions like, “something can go wrong at any minute.” There is a need to assess the quality of care provided for mothers and infants in low-risk labor. We aimed to develop specific quality indicators for low-risk labor care provided primarily by midwives in Japan.

**Methods:**

We used a RAND-modified Delphi method, which integrates evidence review with expert consensus development. The procedure comprises five steps: (1) literature review, including clinical practice guidelines, to extract and develop quality indicator candidates; (2) formation of a multidisciplinary panel; (3) independent panel ratings (Round 1); (4) panel meeting and independent panel ratings (Round 2); and (5) independent panel ratings (Round 3). The three independent panel ratings (Rounds 1–3) were held between July and December 2012.

**Results:**

The assembled multidisciplinary panel comprised eight clinicians (two pediatricians, three obstetricians, and three midwives) and three mothers who were nonclinicians. Evidentiary review extracted 166 key recommendations from 32 clinical practice guidelines, and 31 existing quality indicators were added. After excluding duplicate recommendations and quality indicators, the panel discussed 25 candidate indicators. Of these, 18 were adopted, one was modified, six were not adopted, and four were added during the meeting, respectively.

**Conclusions:**

We established 23 quality indicators for low-risk labor care provided by midwives in labor units in Japan.

## Background

Almost all infants and mothers have high levels of well-being and no serious conditions during term labor. In Japan, care staff sometimes assume that “something can go wrong at any minute during childbirth”, which leads to overprotection against rare adverse events. Midwife-led continuity of care for low-risk labor offers important benefits for mothers and babies, and no adverse effects have been identified [1, 2]. Furthermore, there are regional variations in the numbers of obstetricians, midwives, nurses, and birthing facilities. As such, the quality of labor care is not assured, and performance might vary by facility and provider. However, few quality indicators (QIs) have been developed to measure the quality of care formally. The aim of this study was to develop indicators to measure the quality of low-risk labor care.

The United States Institute of Medicine defines quality as “the degree to which healthcare services for individuals and populations increase the likelihood of desired outcomes and are consistent with current professional knowledge” [3]. The measurement of healthcare quality has gradually diffused into many areas of practice [4]. However, quality assessment is rarely conducted in routine labor and birth, perhaps because most childbirths are normal, without any serious adverse events. Although some indicators exist, their validity is uncertain [5, 6]. Furthermore, there are few such indicators for low-risk labor [7–9]. To measure the quality of care for low-risk labor, the process of care should be examined [10–12].

The majority of deliveries occur following low-risk labor [13]. There is currently no universal definition of low-risk labor; however, it generally includes labor following pregnancies without specific high-risk factors or complications, as detailed in Table 1. We defined low-risk labor by considering three aspects: 1) pregnancy risk self-assessment score; 2) administrative criteria for the payment of medical service fees for high-risk pregnancy and labor administration in Japan; and 3) standards for subjects of low-risk labor care provided by midwives in the clinical practice guidelines (CPGs). When a pregnancy involved any of the items listed in these three categories, we defined it as “not low-risk labor.” Generally, in Japan, an obstetrician decides whether mothers at low risk are capable of giving birth in a midwifery ward. A pregnant female can then choose whether to give birth in an obstetrics ward or a midwifery ward [14–16].

**Table 1:** Definition of low-risk labor^a^

Category	High-risk factors
Physical findings	Age ≥ 40 years, body weight > 80 kg before pregnancy, primiparas with body-mass index > 25% in antepartum
Complications	Thyroid disease, connective tissue disorder, kidney disease, mental disorder, epilepsy, bronchial asthma, neurological disorder, blood-type incompatible pregnancy, hematologic disease, heart disease, uterine cancer, Rh-type blood-group incompatible pregnancy, high blood pressure, pregnancy-induced hypertension, HIV positive, diabetes, gestational diabetes mellitus, antiphospholipid syndrome, pelvic fracture, placenta previa, pregnancy following conization, non-cephalic presentation after 36 weeks’ gestation, premature birth, multiple pregnancy, intrauterine growth retardation, pregnancy following myomectomy, high-grade cervical dysplasia, abdominal surgery other than cesarean section performed or planned during the pregnancy
Pregnancy course	IVF, pregnancy after extensive fertility treatment, undergoing treatment for sexually transmitted disease, risk of mother-to-child transmission, two or fewer pregnancy check-ups, oligohydramnios, polyhydramnios, placenta previa because of previous cesarean section, received definitive diagnosis of fetal malformation or chromosomal abnormalities
History of gynecological diseases	Large uterine fibroids, post-uterine surgery, cesarean section in previous delivery, placental abruption, underwent or plans to undergo abdominal surgery other than cesarean section, cervical incompetency, two or more spontaneous abortions, congenital disease, history of blood-type incompatible pregnancy, eclampsia/HELLP syndrome, gestational diabetes mellitus, stillbirth, neonatal death, delivery of infant <2500 g, severe gestational hypertension ≥160/110 mmHg, history of delivering infant with major malformations

^a^Low-risk labor refers to labor suitable for in-hospital midwifery upon obstetrician approval in the late stages of pregnancy. Specifically, it refers to labor that is expected to result in normal childbirth and excludes the high-risk factors listed above. Items related to abnormalities during labor or after delivery are excluded

In Japan, the numbers of obstetricians, midwives, and maternity facilities vary regionally, with lower numbers generally found in rural areas. In 2008, the Japanese Nursing Association established to promote an inpatient, midwife-led care system as part of the perinatal medical system. This system combines an outpatient department and an inpatient ward, both overseen primarily by midwives, in contrast to regular delivery wards, where obstetricians provide care [17]. Under this system, care is generally provided to mothers undergoing low-risk labor and birth from the beginning of labor until 1 week after birth. If necessary, emergency care is provided by obstetricians in the same hospital [18]. Although QIs of care are needed to evaluate performance and improve care or midwife competency, objective QIs have neither been proposed nor validated within this Japanese system.

In this study, we identified QIs that can be extracted from medical records and applied on site, allowing for visualization and quantification of care quality.

QIs should incorporate the unique aspects of each country’s healthcare system and sociocultural preferences. Accordingly, some countries have established their own QIs [7, 8, 19]. This study aimed to develop QIs for low-risk labor care provided for mothers and infants primarily by midwives in Japan.

## Methods

### Study design

**Fig. 1:**
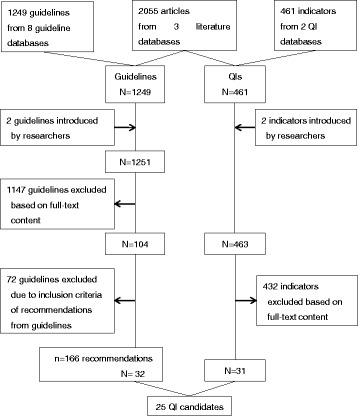
Overview of the literature review: review of the literature, guidelines, and quality indicators (QIs) extracted for generating QI candidates. N, number of extracted clinical guidelines or quality indicators; n, number of extracted recommendations. To avoid duplication, 25 QI candidates were assembled from 32 guidelines and 31 existing QIs

We used a RAND-modified Delphi method (RAND/UCLA Appropriateness Method) [20, 21]. This consensus method has been widely used to develop QIs [22–25]. It comprises two steps: a systematic literature review followed by a face-to-face meeting with a multidisciplinary panel (Fig. 1). Thereby, this method enables the integration of scientific evidence with expert opinions [26]. Because de novo development of evidence-based QIs is very costly and time-consuming, methods using existing CPGs have gained interest as viable alternatives [25]. Thus, rather than searching for primary research articles, we retrieved existing and relevant CPGs and QIs from the literature.

This study was approved by the Ethics Committee of Kyoto University Graduate School and Faculty of Medicine.

### Step 1: Review evidence and generate indicator candidates

To generate QI candidates, we extracted existing recommendations from CPGs related to obstetric care for low-risk labor. In June–August 2012, we searched for data sources using the terms “pregnant women,” “mothers,” “infant,” “perinatal care,” “prenatal care,” “postnatal care,” “delivery,” “obstetric,” and “surgical procedure.” We searched for CPGs in eight databases provided by the following organizations: United States Agency for Healthcare Research and Quality (AHRQ) National Guideline Clearinghouse, Australian National Health and Medical Research Council, Canadian Medical Association, Guidelines International Network, Minds with the Japan Council for Quality Health Care, United Kingdom National Institute for Health and Clinical Excellence (NICE), United Kingdom Scottish Intercollegiate Guidelines Network, and the New Zealand Guidelines Group. Two QI databases (AHRQ National Quality Measures Clearinghouse and National Quality Forum) were used. We also searched medical literature databases, including MEDLINE, CINAHL, and ICHUSHI of the Japan Medical Abstract Society. Finally, we performed a manual search to identify literature that might be relevant to this study.

We searched literature published in English and Japanese between July 2007 and June 2012. We included sets of QI and CPGs for which the title or abstract included the keywords “guideline,” “practice guideline,” “clinical guideline,” “quality indicator,” “clinical indicator,” “performance measurement,” or “quality standard.” We excluded those including the keywords “16 years old and younger,” “40 years old and older,” “premature delivery,” “multiple births,” “breech presentation,” “pre-pregnancy obesity,” “pregnancy complication,” “obstetric history,” “abnormal pregnancy progress,” “infant congenital disease,” “diagnosis and treatment for infant disease,” “birth weight under 2000 g or over 4000 g,” “anesthesia,” “operation and examination procedure,” “28 or more weeks gestation,” “one week post-partum,” or “normal medical care not provided in in-hospital midwifery.” For CPG recommendations, we included graded recommendations expressed as “recommend for,” “recommend against,” and “suggest for.” We did not include recommendations of “weakly suggest,” as indicated by the Grading Recommendations, Assessment, Development, and Evaluations (GRADE) method [27]. We also considered the feasibility of data collection and measurability before inclusion.

Two researchers (KU and SO) extracted data independently. KU assembled the candidates, supervised by two experts: TN, an epidemiologist experienced in developing a variety of Japanese CPGs and QIs, and MK, a nursing instructor and midwife.

### Step 2: Forming a multidisciplinary panel

We assembled a multidisciplinary panel comprising healthcare clinicians (obstetricians, pediatricians, and midwives), public health specialists, and mothers who were nonclinicians. We selected healthcare clinicians with 5 or more years of clinical experience in childbirth and newborn care. We included at least two members from each specialization to prevent disproportionate weight from being placed on particular perspectives. All panel members were required to have either worked or be interested in inpatient midwifery.

Several recently developed QIs in areas other than labor and childbirth have incorporated patient perspectives [28, 29]. We believe that inclusion of mothers’ viewpoints is important for low-risk labor issues. Therefore, we added mothers who were not health professionals to the panel. Potential panel candidates were recruited primarily from staff, students, and graduates of the Kyoto University School of Public Health. After explaining the study context and confirming panel members’ participation, we obtained written informed consent from all panel members. Consequently, the 11 members constituted the multidisciplinary panel.

### Step 3: Independent panel ratings (round 1)

Consensus building involved three rounds of independent rating. During each round, panel members rated the appropriateness of each QI candidate on a 9-point scale, where 1 and 9 were “least suitable” and “most suitable,” respectively. In addition, panel members were given an opportunity to provide comments or suggest additional candidates.

For Round 1, a list of candidate QIs and a description of the rating method were mailed to the panel members. To facilitate decision making, the sources and relevant literature citations for each candidate QI were provided. These ratings were made independently, without interaction among panel members. Based on criteria from the U.S. National Quality Forum Measure Evaluation Criteria [30] and the American College of Cardiology/American Heart Association [31], we rated the appropriateness of each QI according to: 1) usefulness in improving outcomes for mothers and infants; 2) whether the measure is clinically relevant; 3) validity; 4) reliability; 5) feasibility of measure implementation; and 6) overall assessment of the candidate QI (Table 2).

**Table 2:**
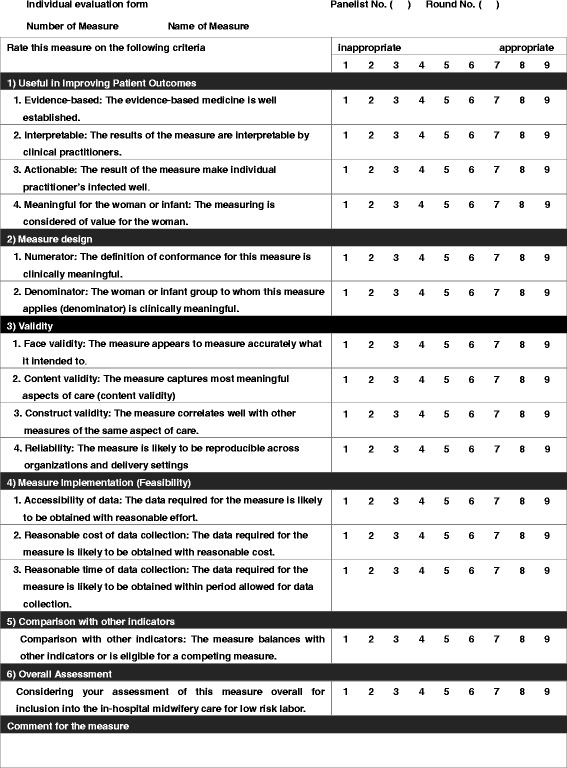
Sample rating questionnaire form

### Step 4: Panel meeting and independent panel rating (round 2)

In Round 2, a face-to-face meeting moderated by KU and TN was held. Each member received a document showing the distribution of Round 1 ratings by all members. In this meeting, candidate QIs were discussed, and decisions regarding adoption were made. During the discussions about the QI candidates, each panel member rated them on a questionnaire. Additional candidate QIs were also proposed at this meeting.

### Step 5: Independent panel rating (round 3)

In Round 3, the additional candidate QIs were evaluated using a second postal survey (conducted in the same manner as the first) to determine which QIs would be adopted (see below).

### Statistical methods

QI candidates were adopted if the median individual “overall assessment” during Round 2 or 3 was greater than 7 and if three or fewer panel members rated it less than 3 [20].

## Results

From the literature review, we extracted 32 CPGs (166 recommendations) and 31 existing QIs (Fig. 1). We selected 16 guidelines from the AHRQ National Guideline Clearinghouse, 8 from NICE Guidance, 3 from the Canadian Medical Association, 2 from Minds with the Japan Council for Quality Health Care, 1 from the Australian National Health and Medical Research Council, 1 from the Guidelines International Network, and 1 obtained from our manual search. Twenty-five QI candidates pertaining to the Japanese healthcare system were developed from the CPGs and QIs.

All panel members agreed to participate in the study. All responded to the postal surveys, attended the meeting, and participated in the entire process. The consensus-development process was completed in December 2012.

**Fig. 2:**
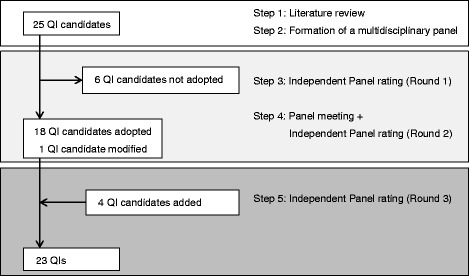
Development process of quality indicators using a RAND-modified Delphi method for low-risk labor care. This *flow diagram* illustrates each stage within the process of quality indicator development. The *top white box* indicates the identification of initial quality indicator candidates: Steps 1–2. The middle gray square indicates the first rating of quality indicator candidates: Steps 3–4. The *lower dark gray square* indicates the final stage, in which the panel rated additional quality indicator candidates: Step 5

Figure 2 shows the process of QI development. The Round 2 ratings resulted in 18 QIs selected from among the 25 QI candidates; 6 indicator candidates were not adopted. One indicator was modified and included as an additional candidate following the panel’s suggestion that it was needed for induced labor and to promote delivery (Indicator 9, Table 3). Four new QI candidates were introduced in the panel meeting (Indicators 2, 12, 14, and 23, Table 3). Five indicator candidates proposed at the meeting were ultimately adopted at Round 3. Consequently, 23 QIs were established (Table 3).

**Table 3:** Quality indicators for low-risk labor care provided primarily by midwives in Japan

No. Indicator	Rating result	Median	Agreement (%)^b^
1. Primipara who has enrolled in a childbirth class about antenatal care and delivery by 36 weeks gestation	adopted	8	8 (72.7%)
2. Discussed a birth plan^a^	added and adopted	9	11 (100%)
3. Initial assessment of labor risk at admission	adopted	7	10 (90.9%)
4. Assessment during first stage labor	adopted	8	8 (72.7%)
5. Assessment during second stage labor	adopted	9	10 (90.9%)
6. Women with a term, singleton infant in vertex position delivered by Cesarean section	adopted	8	10 (90.9%)
7. Women with a term, singleton infant in vertex position delivered by Vaginal delivery	adopted	9	11 (100%)
8. Women with a term, singleton infant in vertex position delivered by Instrument delivery	adopted	9	11 (100%)
9. Women with a term, singleton infant in vertex position delivered by labor induction^a^	modified and adopted	8	9 (81.8%)
10. Term infant with Apgar score less than 7 at five minutes after birth	adopted	9	9 (81.8%)
11. Living infant with birth injuries	adopted	7	9 (81.8%)
12. Respiratory support: Resuscitation for asphyxiated term neonate with low oxygen concentrations and oxygen saturation measured by pulse oximetry immediately after birth^a^	added and adopted	8	10 (90.9%)
13. Women with perineal tear and no episiotomy	adopted	9	11 (100%)
14. Second degree perineal laceration^a^	added and adopted	8	9 (81.8%)
15. Third or fourth degree perineal laceration	adopted	8	11 (100%)
16. Postpartum hemorrhage more than 500 g within 2 h of birth	adopted	8	10 (90.9%)
17. Infant admission to pediatrics department within a week after birth (excludes those with congenital anomalies)	adopted	8	9 (81.8%)
18. Infants that were fed only breast milk at the time of discharge from the hospital	adopted	8	11 (100%)
19. Peer review of severe adverse events with medical staff	adopted	8	9 (81.8%)
20. Woman switched to receive care provided primarily by obstetricians from midwifery ward	adopted	8	11 (100%)
21. Mother received cessation counseling intervention (including guidance on smoking cessation) if identified as either a tobacco user or passive smoker	adopted	7	6 (54.5%)
22. Infant administered vitamin K three times by one month after birth	adopted	9	11 (100%)
23. Infants who had been fed only breast milk at the time of the health examination for children of 1 month of age^a^	added and adopted	9	11 (100%)
24. Women with second degree perineal laceration, not due to instrument delivery	not adopted	1	4 (36.4%)
25. Women that unintentionally retained foreign objects during labor and delivery	not adopted	5	4 (36.4%)
26. Neonatal bloodstream infections within 48 h of birth	not adopted	5	2 (18.2%)
27. Medication error made in non-recommended abbreviations, symbols or dose designations used in medical prescriptions	not adopted	1	0 (0%)
28. Women with complex social factors who were offered additional support and information on public resources	not adopted	2	1 (9.1%)
29. Women that received antenatal or postnatal guidance regarding body weight and physical activity	not adopted	6	5 (45.4%)

These indicators denote the frequency with which care was provided and recorded for women admitted to an in-hospital midwifery ward

^a^These indicators were advanced in Step 4 and rated in Step 5

^b^Agreement (%) indicates the proportion of members who gave ratings of 7–9 points to adopt a candidate quality indicator

## Discussion

We established 23 QIs for low-risk labor care provided primarily by midwives using a RAND-modified Delphi method based on CPGs and existing QIs. The main purpose of using QIs for low-risk labor care is to ensure effective quality improvement of providers’ performance.

Our study differs distinctly from previous studies, which have reported QIs for low-risk labor or normal birth [7–9] but did not report transparent procedures for how they created QI candidates following their literature searches. Only one study reported evidence sources, including guidelines. Furthermore, these studies considered both home and hospital birth. We focused on low-risk labor care provided primarily by midwives in hospital.

Panel composition may affect the final consensus on QIs. While incorporating patient views is considered important, patient participation in QI development has been limited [24, 29, 32]. Our panel included three mothers with birth experience, so the QIs we developed may more readily reflect insight and ideas from mothers concerning the quality of care in inpatient midwifery wards.

Prior attempts at QI development have widely used the RAND-modified Delphi method (i.e., the RAND/UCLA appropriateness method) [22, 25, 33], whereby a literature review is conducted and a consensus is developed. Methods to extract guidelines during the literature review have attracted much attention, because the traditional review method using primary research articles is time-consuming. However, such methods have only recently been developed, and thus, they have yet to be standardized [24, 25]. Processes to develop new QIs based on existing CPGs have been described in various forms and therefore may include a degree of arbitrariness. Thus, to increase the reproducibility of the present study, two of the authors (KU and SO) independently extracted evidence under the supervision of another author (TN). When developing guideline-based QIs, objective and transparent processes must be executed, even if some of their aspects may remain arbitrary. As in traditional systematic reviews, QI developers should describe their methods and reasons for selecting recommendations from existing CPGs and clarify which databases, selection criteria, and search keywords were used. One challenge we faced was determining the grades for CPG recommendations. The present study used all CPG recommendations except “suggest not to do” or “weak recommendation against.” These expressions are sometimes difficult to use when interpreting whether a recommendation should be implemented [27].

### Strengths and limitations

Our results should be considered in light of some limitations. First, panel composition may influence consensus development and outcomes at several points. If a member has expertise in a certain area and makes strong assertions, the consensus may become biased. To address this concern, we had two moderators (KY and TN) and two moderator assistants (SO and MK) to prevent one-sided discussions. At the beginning of the meeting, the moderators explained the rules for discussion (e.g., “respect the end time of the meeting”). In addition, one assistant managed the meeting time, while the other confirmed each panel member’s degree of participation, reporting observations to the moderators. Neither the moderators nor the assistants voted in the consensus development.

Second, our electronic search did not identify some CPGs, e.g., the WHO guidelines “Making Pregnancy Safer WHO 2009” and “Care in Normal Birth WHO 1996”. The former mainly included indicators to assess structure of facilities, however, we focused on the process and outcome indicators for each individual practice. The latter was not relevant considering our planned publication period and the main contents are covered by the following CPGs that we included. Although including these CPGs would not seriously change the present results, search strategy and selection criteria of existing CPGs may need refinement when updating the current QIs.

Third, the validity of our consensus method may be influenced by the representativeness of the panel members. Complete representativeness is probably not possible, but transparency in how the panel is assembled and of the whole consensus process is critical for readers to assess the validity of the outputs. We have detailed the selection criteria, recruitment process, and panel characteristics. The panel included only one member with experience in CPG development, whereas guideline developers and representatives of academic societies might be better qualified for QI development than others, because they are expected to be well informed in these areas. When relevant academic societies develop QIs based on their own clinical practice guidelines, our proposed QIs might guide their activities. The present QI sets need practical validation to confirm their clinical relevance [26]. A study to test their validity is currently in progress.

Fourth, patients’ perspectives may not have been adequately included. The 23 QIs developed in this study did not include items assessing such factors as patient experience, patient–provider relationship, and its empowerment of mothers. This may be a general limitation of QI development based on guidelines. Although patient representatives were included in the panel, patient experience and the patient–provider relationship were not reflected in the QI items.

Ideally, QI development should include the patient’s viewpoint, but this is rare in reality. Therefore, a strength of our study was the inclusion of individuals who had experienced labor but were not healthcare professionals [24, 34]. There is a danger that nonclinicians may have difficulty rating indicator candidates and joining the discussion. To address this, we provided additional information to explain specialized terms pertaining to the list of QI candidates. We also carefully addressed their questions by mail or telephone throughout the entire process. As the three panel members who were not healthcare professionals were healthcare researchers, there may have been fewer barriers to their participation in the consensus process than that of a general layperson. It was found that they contributed sufficiently to the discussion.

## Conclusions

Using a RAND-modified Delphi method incorporating CPGs and existing QIs, we established 23 QIs for low-risk labor care provided primarily by midwives. These QIs can be used to assess and improve practices for low-risk labor managed by midwives. They should also initiate discussion of QIs among relevant health professionals and societies.

## Abbreviations

AHRQ: Agency for Healthcare Research and Quality
CPG: Clinical practice guideline
NICE: National Institute for Health and Clinical Excellence
QI: Quality indicator
